# Fine-grained few-shot class-incremental identification of medicinal plants via frequency-aware contrastive learning

**DOI:** 10.3389/fpls.2026.1730047

**Published:** 2026-02-13

**Authors:** Chaoqun Tan, Zhonghan Qin, Zihan Tang, Yongliang Huang, Ke Li

**Affiliations:** 1 School of Intelligent Medicine, Chengdu University of Traditional Chinese Medicine, Chengdu, China; 2 National Key Laboratory of Fundamental Science on Synthetic Vision, School of Computer Science, Sichuan University, Chengdu, China; 3 School of Economics, Southwestern University of Finance and Economics, Chengdu, China; 4 Department of Pharmacy, Hospital of Chengdu University of Traditional Chinese Medicine, Chengdu, China

**Keywords:** contrastive learning, fine-grained few-shot incremental learning, frequency-aware, identification, medicinal plant

## Abstract

Developing robust algorithmic tools for accurately identifying diverse medicinal plant species is critical for advancing precision medicine. Although deep learning methods have shown considerable promise, they generally require large-scale annotated datasets, which are often difficult to acquire given the vast taxonomic diversity and limited labeled samples available for many plant species. To address this, we propose a novel Frequency-Aware Guided Domain Enhancement Contrastive Learning (FGDE) framework, designed to incrementally learn new categories from few annotated examples while alleviating catastrophic forgetting and overfitting. Our approach integrates high- and low-frequency components to refine feature representations, using multi-frequency fusion to preserve detail-enhanced information. Contrastive learning is further employed to strengthen multi-semantic aggregation and extract discriminative features across both visual and label domains. Additionally, we introduce a multi-objective loss function to enhance semantic compactness within base classes and improve separation among incremental classes. Extensive experiments demonstrate that FGDE significantly outperforms state-of-the-art methods on our collected dataset and two public benchmarks. These results underscore the potential of our model to support practical applications in intelligent plant identification and precision agriculture.

## 1 Introduction

Medicinal plants, renowned for their therapeutic properties and historical significance, play a pivotal role in the clinical practice of traditional medicine (Sun et al., 2022; Zang et al., 2025). Consequently, they have garnered significant attention from both traditional healers and modern medical practitioners (Wang et al., 2020; Armijos et al., 2022; Chen et al., 2025). However, due to the confusion by different varieties for the affected quality and commercial value that have been reported, increasing concern has been expressed by the public (Xiao et al., 2022; Zhang et al., 2022; Vani et al., 2025). Therefore, accurate authentication of medicinal plant species is critical for practical application. Conventionally, detecting active ingredients such as organic acids and flavonoids serves as the gold standard for identifying medicinal plant varieties (Wu et al., 2025). While these laboratory-based methods offer high precision, they are often time-consuming, costly, and reliant on specialized equipment (Fitzgerald et al., 2020; Xiao et al., 2025). Alternatively, intelligent sensory technologies combined with chemometric methods have gained traction, yet they remain constrained by specific instrumentation requirements.

With recent advancements in Deep Learning (DL), computer vision has emerged as a promising, non-destructive, and rapid solution for plant identification, demonstrating remarkable success in medical image classification (Pandey and Jain, 2022; Huang et al., 2025; Wang et al., 2025). The efficacy of DL-based approaches in automating taxonomy is widely acknowledged (Attri et al., 2023). However, these data-driven models typically rely on large-scale annotated datasets to learn robust feature representations (Wang et al., 2021). In the context of medicinal plants, the sheer diversity of species renders the construction of comprehensive, large-scale annotated datasets impractical. Furthermore, acquiring images across a broad spectrum of varieties presents significant challenges due to the inherent difficulties in sample collection (LeCun et al., 2015; Tan et al., 2024). Consequently, how can we design a model capable of effectively learning feature representations from limited annotated data? Developing a system that can rapidly adapt to new concepts using only a few annotated samples would be highly beneficial for the advancement of the field.

Few-Shot Learning (FSL) (Fei-Fei et al., 2006; Gao et al., 2023) aims to enable image classification models to adapt to new tasks using scarce annotated samples. These frameworks typically involve a training phase for model adaptability and an adaptation phase for new tasks (Dvornik et al., 2019). Several studies have successfully applied FSL to plant analysis, such as leaf classification (Argüeso et al., 2020), plant detection (Rezaei et al., 2024), and hyperspectral categorization (Cai et al., 2023). However, standard FSL methods are prone to catastrophic forgetting, where adapting to new tasks degrades performance on previous ones. To mitigate this, Few-Shot Class-Incremental Learning (FSCIL) (Tao et al., 2020) was introduced, utilizing techniques like neural gas networks (Martinetz and Schulten, 1991; Prudent and Ennaji, 2005) to dynamically model feature space topology. Despite this progress, mainstream approaches (Ahmed et al., 2024; Han et al., 2024) often employ a frozen backbone pre-trained with cross-entropy loss. This strategy frequently fails to effectively separate class margins, leading to poor generalization (Raichur et al., 2024; Zhou et al., 2024). Moreover, the data often presents fine-grained features: minimal distinction between different species (low inter-class variance) and significant variation within the same species (high intra-class variance). Such ambiguity hinders the model’s ability to discriminate between new and old classes, resulting in false classifications.

Most existing techniques in fine-grained classification primarily focus on extracting image edge signals or high-frequency features (Song et al., 2023). While these detailed features are generally effective in revealing subtle inter-class differences, it is crucial to further enhance the distinction between fine-grained classes. Subsequently, it is essential to enhance the distinction between fine-grained classes and achieve clearer clustering of novel and old data, even with limited samples.

**Figure 1:**
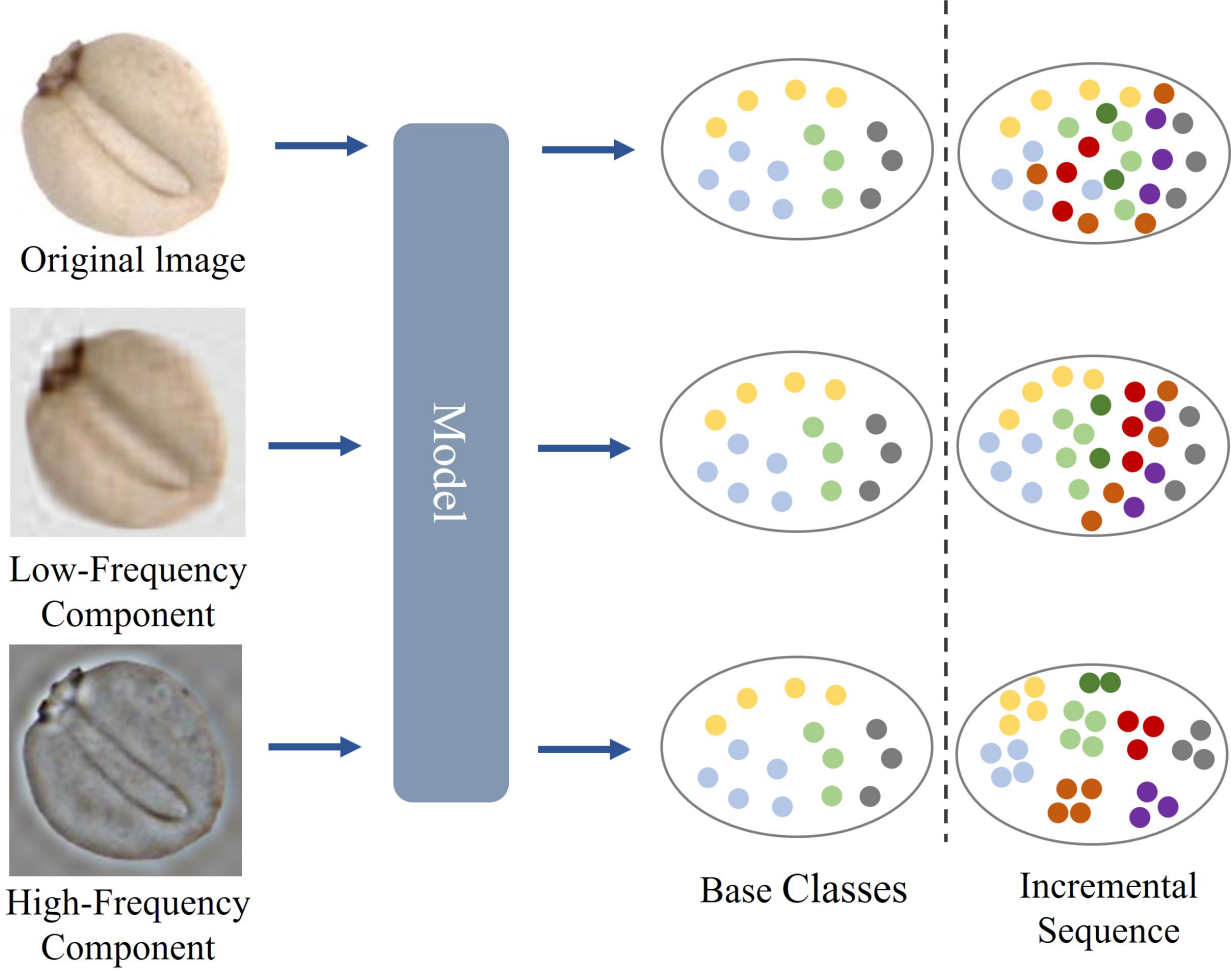
Illustration of our FGDE. Different classes are marked in different colors. Our proposed network extracts the high-frequency and low-frequency features using the Discrete Cosine Transform (DCT). Enhanced features improve the clustering performance of the model.

Motivated by these challenges, this paper proposes a novel Frequency-Aware Guided Domain Enhancement Contrastive Learning Model (FGDE). This framework constructs discriminative features by integrating high- and low-frequency components and leverages the class-clustering capability of contrastive learning. The result is a feature distribution characterized by improved intra-class compactness and inter-class separability. As illustrated in **Figure 1**, the detailed features are refined by incorporating high-frequency components to enhance domain-specific representations (Li et al., 2023). The proposed method is described in the third section, and the experimental results and analysis are shown in the fourth section. The main contributions of this paper are summarized as follows:

1. A novel Frequency-Aware Guided Domain Enhancement Contrastive Learning Model (FGDE) is proposed to strengthen the fine-grained semantic extension of base classes and the separation of subsequent classes. It achieves detail-enhanced feature representation by integrating multi-frequency components, thereby refining domain-specific distinctions.
2. We propose high-frequency and low-frequency components to enrich the original features and unearth class-discriminative information in both the visual and label domains. Subsequently, it enhances multi-semantic aggregation awareness, facilitating more precise differentiation of fine-grained images.
3. We introduce contrastive loss, cross-entropy loss, and feature augmentation loss. This mechanism minimizes intra-class variance while maximizing inter-class separation, significantly enhancing the model’s discriminative power and generalization capabilities.
4. We showcase robust performance on our datasets and public datasets, outperforming previous state-of-the-art methods. Furthermore, we perform a thorough analysis to evaluate the importance of each component.

## 2 Data collection and preprocessing

### 2.1 Sample preparation

We collected 28 different specimens and their derived products, which all samples were sourced from the Lotus Pond Chinese Medicinal Plant Market in Chengdu China. These samples were authenticated by experts from the Chengdu Institute of Food and Drug Control (Chengdu, China). The dried samples were obtained from the original intact specimens. Post-collection, they were stored in standard cold storage conditions.

### 2.2 Data acquisition

**Figure 2:**
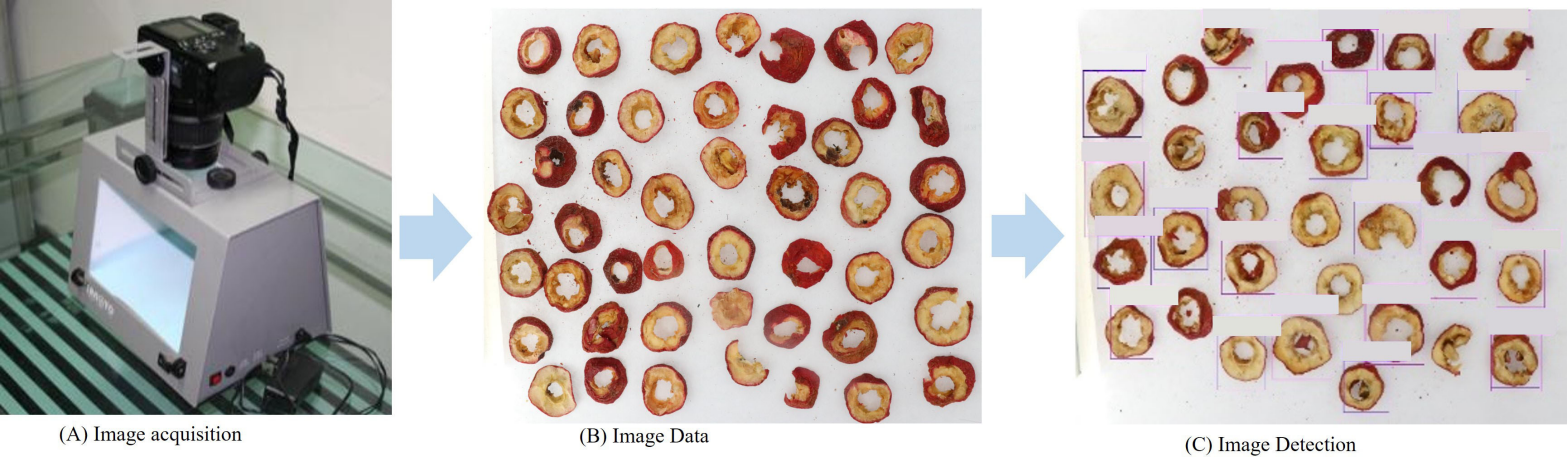
The image detection to detection results. **(A)** Image Acquisition, **(B)** Image Data, **(C)** Image Detection.

A self-developed high-resolution data acquisition device (Canon EOS 60D) was used to acquire images in **Figure 2A**. The device is composed of a box, a light system, and an image acquisition system, which can provide stable and consistent environmental conditions. The image acquisition process is illustrated in **Figure 2**.

**Figure 3:**
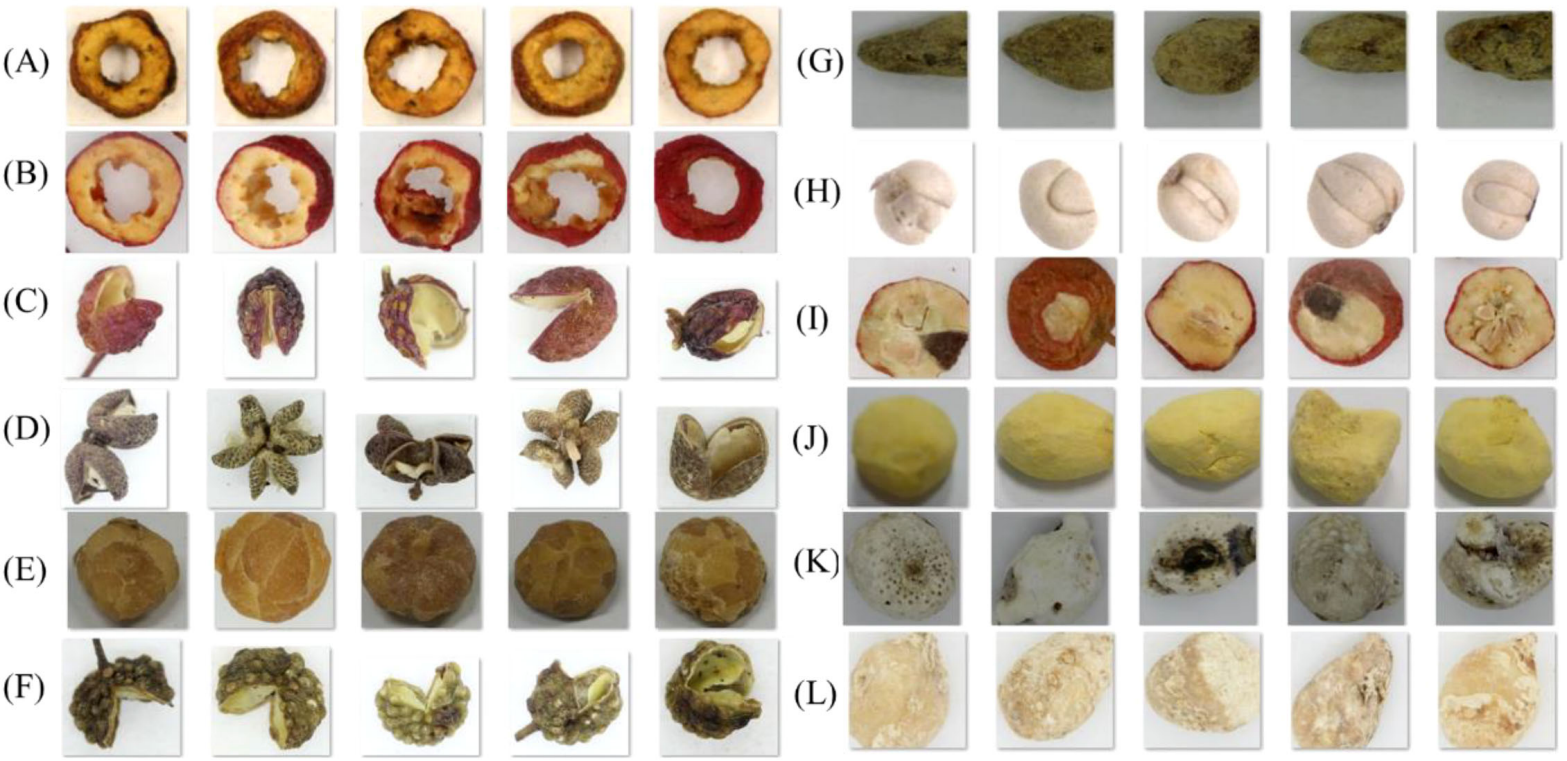
Random samples from the dataset, which consists of 28 different CHMs and their produced products. Namely **(A)** chaoshanzha **(B)** honghuajiao **(C)** jiaoshanzha **(D)** hanyuanhuajiao **(E)** shanzhatan **(F)** qingjiao **(G)** sichuanhuajiao **(H)** tengjiao **(I)** jiangbanxia **(J)** lubei **(K)** songbei **(L)** shengbanxia.

All images are captured using a 35mm CMOS sensor with a resolution of 5120×3840, as shown in **Figure 2B**. Images are annotated and cropped to obtain a target, see **Figure 2C**. We remove incomplete, blurry, and inappropriate images. Our collected dataset is shown in **Figure 3**. Due to the potential for a highly unbalanced training dataset, such as the interfered classes within our dataset. This issue is addressed by balancing each class through data augmentation. Specifically, we augment the data to ensure a uniform distribution of 250 samples in each class.

Different processed plants of *Shanzha* include *Chaoshanzha*, *Jiaoshanzha*, and *Shanzhatan*. Similarly, various processing plants are used to obtain different processes with Banxia, including *Jiangbanxia*, *Fabanxia*, *Qingbanxia*, and *JingBanxia*. However, *ShuiBanxia* is often used as a counterfeit product of *Qingbanxia*. Additionally, Jiang *Nanxing*, as a processed product derived from Tiger’s Paw Southern Star, is commonly considered a fake of *Jiangbanxia* in the commercial market. *Lubeimu*, *Qingbeimu*, and *Songbiemu* are the most common circulation of *ChuanBeimu*. According to the properties of images, all data are detected to remove redundant pixels that contain no information.

## 3 Methods

### 3.1 Problem definition

Continuous incremental classes are the key factors in FSCIL. In this paper, the first session can learn a generalizable representation. Then, multiple few-shot incremental classes are executed. There is the training data $D_{t}^{i}={\left\{\left(x_{i},y_{i}\right)\right\}}_{i=0}^{N_{t}}$ is the training data from session t, $x_{i}$ and $y_{i}$ are the i-th image and corresponding label respectively. The training images are expressed as $D_{s}=\left\{D^{0},D^{1}\ldots .D^{N}\right\}$. For the initial sequence ${\text{D}}^{0}$, the image domain contains $C^{0}$ classes, and the label domain is $L^{0}$. For subsequent incremental sequences, the label domain has no overlap, the rest contained in new classes are invisible in base data. When the $D_{train}^{i}$ are trained, and the model is tested in ${\text{D}}_{\text{test}}^{\text{i}}$, which contains all encountered classes $C_{0}\cup \;C_{1}\cup \;\ldots .C_{t}$ in the t-th subsequent. In FSCIL, the initial sequence is with many samples, and the model only has access to a few samples in the following subsequent. Specifically, the incremental data are always organized in N-way K-shot format, N is the class, and K represents the training images of each class.

### 3.2 Overview

**Figure 4:**
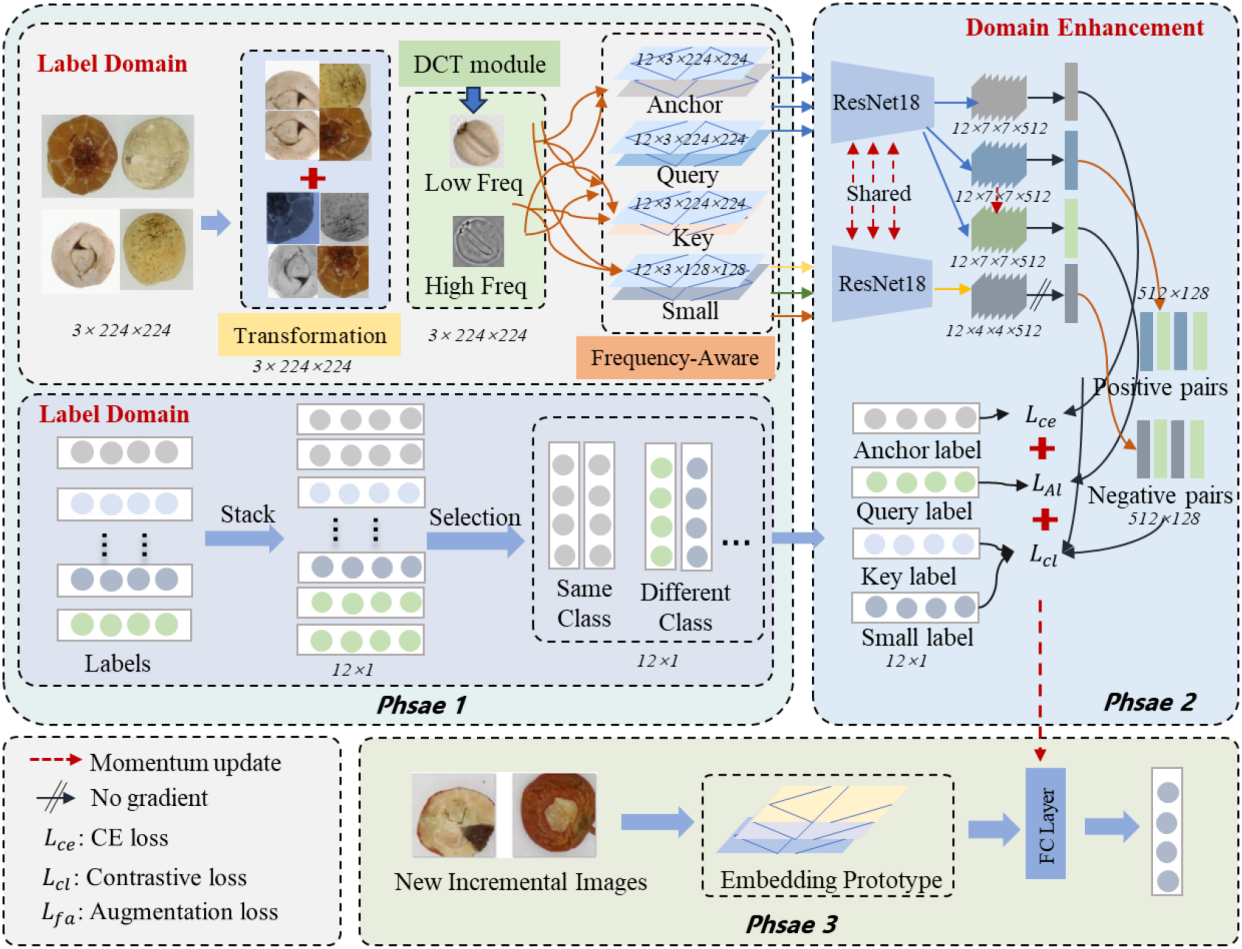
The overall pipeline of our FGDE framework consists of three phases. Phase 1 emphasizes learning richer representations of the original space for both the image and label domains through multiple predefined transformations. Phase 2 involves leveraging contrastive learning to distinguish between positive and negative sample pairs. Phase 3 focuses on training the limited new classes to mitigate catastrophic forgetting and reduce overfitting.

The architecture of our proposed FGDE model is illustrated in **Figure 4**. In the first phase, multiple predefined augmentations are applied to enrich the fine-grained images. Subsequently, Discrete Cosine Transform (DCT) is employed to extract multi-frequency features, which are fused with the original images to construct high-frequency and low-frequency enhanced representations. Simultaneously, label representations are expanded to encapsulate the semantic consistency of the images. The visual patches and these expanded labels interact to ensure cross-modal alignment and refine the embedding space, thereby improving the separability of base classes. In the second phase, generated contrastive learning pairs are utilized to enhance multi-semantic aggregation and mine class-discriminative information. Here, semantic granularity is enriched via contrastive learning. We jointly optimize contrastive, feature augmentation, and cross-entropy losses to minimize intra-class variance and maximize inter-class distance. During the third phase, the model adapts to new classes using limited few-shot samples. A similar metric is employed to assign incoming samples to their respective prototypes, ensuring robust generalization and stability while mitigating catastrophic forgetting.

#### 3.2.1 Frequency-aware guided multi-semantic feature enhancement

Data scarcity in base classes restricts the diversity of learned semantic features, leading to poor generalization and unclear class boundaries. To enhance feature robustness, we apply targeted visual transformations, focusing on color and shape as suggested by previous studies. Specifically, we employ random cropping to expand the fine-grained feature space. Given an image X, the cropping dimensions are defined as $w_{crop}∼\text{Rand}\left(w_{min},w_{max}\right)$ and $h_{crop}∼\text{Rand}\left(h_{min},h_{max}\right)$. Given a rand point ( $x_{r}$, $y_{r}$), where $x_{r}\in \left[0,\;w-w_{crop}\right]$, $y_{r}\in \left[0,\;h-h_{crop}\right]$. Thus, the point ( $x_{f}$, $y_{f}$) of the lower right corner of the final cropping area are computed in Equation 1:

(1) $$\{\begin{array}{c} x_{f}=x_{r}+w_{crop}-1\\ y_{f}=y_{r}+h_{crop}-1 \end{array}$$

Random cropping of varying sizes is employed to capture local information, enhancing both local feature understanding and fine-grained semantic perception. To further enrich class-aware semantics, we introduce a transformation set $ℱ\in \left\{T_{c},T_{r}\right\}$, consisting of color jittering ( $T_{c}$) and random rotation ( $T_{r}$). The processed RGB images are then transformed into the frequency domain using the 2D DCT, which expresses pixel data via a linear combination of cosine basis functions. Leveraging the superior energy compaction of DCT over the complex-valued DFT (He et al., 2020; Huang et al., 2025), each channel of the input image X is converted to the frequency spectrum $P^{2d}$ in Equation 2:

(2) $$P_{h,w}^{2d}=\alpha_{h}\alpha_{w}\sum_{a=0}^{H-1}\sum_{b=0}^{W-1}\cos\left(\frac{\pi h}{H}\left(a+0.5\right)\right)\cos\left(\frac{\pi w}{W}\left(b+0.5\right)\right),$$

where h∈{0,1,…H−1}, w∈{0,1,…W−1} represent the horizontal and vertical frequency indices. The normalization coefficients $\alpha_{h}$ and $\alpha_{w}$ are defined as in Equation 3:

(3) $$\alpha_{k}=\{\begin{array}{cc} \sqrt{1/N}, & \text{if }k=0\\ \sqrt{2/N}, & \text{otherwise} \end{array},\text{where }N\in \left\{H,W\right\}\;$$

In the resulting spectrum $P^{2d}$, low-frequency components are concentrated near the origin (0,0), while high-frequency components are distributed in the peripheral regions. Then, we apply a binary mask M to separate the spectrum into low-frequency and high-frequency components. We define a cut-off threshold τ based on the Manhattan distance in the frequency domain. The mask M is defined as in Equation 4:

(4) $$M_{h,w}=\{\begin{array}{cc} 1, & \text{if }h+w\leq \tau \\ 0, & \text{otherwise} \end{array}$$

Subsequently, the low-frequency spectrum $P_{low}^{\left(2d\right)}$ and high-frequency spectrum $P_{high}^{\left(2d\right)}$ are derived via the Hadamard product ( ⊙): $P_{low}^{\left(2d\right)}=P^{\left(2d\right)}⊙M,P_{high}^{\left(2d\right)}=P^{\left(2d\right)}⊙\left(1-M\right)$. Finally, we project the masked spectra back to the spatial domain using the 2D Inverse DCT (IDCT) in Equation 5:

(5) $${\tilde{I}}_{h,w}^{2d}=\sum_{a=0}^{H-1}\sum_{b=0}^{W-1}\alpha_{h}\alpha_{w}P_{h,w}^{2d}\cos\left(\frac{\pi h}{H}\left(a+0.5\right)\right)\cos\left(\frac{\pi w}{W}\left(b+0.5\right)\right)$$

These components are then sent into the encoder and obtain the original image $I_{X}$, low-frequency $I_{l}$ and high-frequency $I_{h}$ feature maps. This enables the extraction of discriminative details from high-frequency components and structural context from low-frequency components. The feature extraction is defined as in Equation 6:


$$I_{l}^{\prime }=f_{\theta }\left(I_{l}\times X+X\right)$$

(6) $$I_{h}^{\prime }=f_{\theta }\left(I_{h}\times X+X\right)\;$$

Utilizing enhanced discriminative feature maps as prior knowledge augments the model’s ability to capture critical information and adapt to incremental data. Specifically, by aligning samples with class prototypes via the high-frequency features $I_{h}^{\prime }$, we encode fine-grained details that effectively sharpen decision boundaries and enhance model performance. Then, the embedded image is computed by Equation 7:

(7) $$I=f_{DCT}\left(f_{{\text{T}}_{\text{r}}}\left(f_{{\text{T}}_{\text{c}}}\left(C_{0}\right)\right)\right),\;C_{0}=\left\{X,I_{h},I_{l}\right\}$$

Where $C_{0}$ is designated as the encountered class. This can extend various semantics and fill the unallocated image embedding space. It can also provide semantic knowledge, which encourages extensive learning of different semantics for better generalization.

For label domain, the predefined alternations can generate multiple augment image-label pair ( x, y), where $ℱ\left(x,\;y\right)={\left\{x_{n},\;y_{n}\right\}}_{n=1}^{N}$. N is the number of transformed extension space. $x_{n}$ are the generated extension images, and the corresponding labels is $y_{n}=y\times N+n$. Thus, the label space is extended with the fine-grained class-aware embedding derived from the original space. The association between the image domain and the label domain can effectively provide richer semantic details to improve the accuracy. Likewise, the training within the embedding space ℱ can be expressed by Equation 8:

(8) $$L_{class}\left(f;x,y\right)=\frac{1}{N}\sum_{i=1}^{N}l_{ce}\left(f\left(x_{n}\right),\;y_{n}\right)$$

#### 3.2.2 Detail-enhanced discriminative feature representation

Although effective for coarse classification, existing methods are limited in handling fine-grained data. We therefore propose an embedding-based supervised contrastive learning strategy using the MoCo (He et al., 2020) framework. This method optimizes feature distances by clustering positive pairs and separating negative ones. Given an instance (x,y), we generate a query view $x_{q}={\text{Aug}}_{q}\left(x\right)$ and a key view $x_{k}={\text{Aug}}_{k}\left(x\right)$ via data augmentation. A shared encoder $f_{q}$, comprising a feature extractor and a classifier, is then employed to extract the corresponding features. As shown in Equation 9:

(9) $$f_{q}=\omega^{T}f\left(x\right)$$

where $\omega^{T}\epsilon {\mathbb{R}}^{\text{d}\times \left|{\text{C}}_{0}\right|}$ is the weight value, and $f\left(x\right)\epsilon {\mathbb{R}}^{\text{d}\times 1}$ is the feature function. Among them, the query encoder $f_{q}$ are encoded through gradient descent, while the key encoder $f_{k}$ are encoded by a progressing encoder, driven by a momentum update with the $f_{q}$. The queue of key embedding is maintained to store the feature vector.

In the label domain, a label queue maintains labels corresponding to the feature queue, facilitating the differentiation of positive and negative pairs. This queue preserves an identical length to the feature queue. Subsequently, the contrastive loss is computed to drive the model to capture discriminative fine-grained features. This optimization effectively minimizes intra-class distance while maximizing inter-class variation, thereby fostering deep interaction between the visual and label domains.

##### 3.2.2.1 Inter-class variation

Usually, denote the representations of a class for a gathering center as prototypes $P_{j}$, all prototypes of different classes are far away from each other. The $P_{j}$ is expressed by Equation 10:

(10) $$P_{j}=\frac{1}{N_{j}}\sum_{a=1}^{N_{j}}X_{a}$$

where $N_{j}$ is the number of classes j. ${\text{X}}_{\text{a}}$ is the feature vector of the a-th sample. Thus, denoted two prototypes $P_{j}$ and $P_{k}$ for class j and k in base session, the Euclidean distance of inter-class variation is calculated by Equation 11:

(11) $$d_{j,\;k}^{inter}=\sqrt{\sum_{d}{\left(P_{j}^{i}-P_{k}^{i}\right)}^{2}}$$

Where d is the dimension of the feature vector. i is the i-th sample. For the subsequent incremental sequence, the novel classes are also obtained by computing the distance between their prototypes with the samples.

##### 3.2.2.2 Intra-class distances

The analysis of intra-class distances involves computing the Euclidean distances between the samples and prototype $P_{j}$ within the same class j, and then determining the average value. For the testing sample, the intra-class distances are computed by Equation 12:

(12) $$d_{j}^{intra}=\frac{1}{N_{j}}\sum_{a=1}^{N_{j}}\sqrt{\sum_{d}{\left(x_{j}^{i}-P_{j}^{i}\right)}^{2}}$$

Where $x_{j}^{i}$ is the feature vector of samples. The smaller the intra-class distance, the more effectively the samples within the same classxcluster. This enhances the distinct separation of local information in the feature space and is crucial for accurate fine-grained identification.

##### 3.2.2.3 Augmentation feature analysis

The model should also focus on global features for the multi-transformation imbalanced-grained data. To improve the generalization ability of class separation, we consider the global augmentation set from the generalization features. It serves as query view and optimizes the feature space by learning the general features of different classes. Likewise, the image-label pairs $\left\{x_{m},\;y_{m}\right\}$ for global augmentation can be processed by Equation 13:

(13) $$L_{Al}\left(f;x,y\right)=\frac{1}{M}\sum_{i=1}^{M}l_{ce}\left(f\left(x_{m}\right),\;y_{m}\right)$$

With the augmentation feature analysis, the model can better concentrate the detail-enhanced information to distinguish imbalanced fine-grained images and optimize the feature space.

#### 3.2.3 Incremental class inference

With the incremental sequence, the backbone network is fixed, and the classifier is computed by computing the novel class prototypes. The novel class information can be acquired to enable the extension of classifiers with the prototypes of basic classes and extended-augmentation classes. As shown in Equation 14:

(14) $$W_{N}^{class}=\left\{w_{11}^{0},w_{12}^{0},\ldots w_{bN}^{0}\right\}\cup \;\left\{w_{11}^{1},w_{12}^{1},\ldots w_{bN}^{1}\right\}\ldots \cup \;\left\{w_{11}^{t},w_{12}^{t},\ldots w_{bN}^{t}\right\}$$

where b is the number of basic classes, N is the number of transformed extension space, and t is the number of incremental sequences. The prototypes ${\text{W}}_{\text{n}}$ represents the focus of global and local fine-grained semantics from the original classes, shown in Equation 15:

(15) $$W_{n}=\left\{w_{1n}^{0},w_{2n}^{0},\ldots w_{bn}^{0}\right\}\cup \;\left\{w_{1n}^{1},w_{2n}^{1},\ldots w_{bn}^{1}\right\}\ldots \cup \;\left\{w_{1n}^{t},w_{1n}^{t},\ldots w_{bn}^{t}\right\}$$

Subsequently, the classifier is updated by the novel classes’ prototypes combined with the original class prototypes. It can help push the novel samples away from the distributions of the old classes and benefit novel class-aware semantic information generalization. The FC layer of the model is updated by contrasting novel query samples with these slowly evolving key embedding of base classes from the feature queue. Finally, the cosine similarity between embedding and all prototypes is computed to obtain the inference results for the test image. It can be formulated as Equations 16 and 17:

(16) $$Pred=argmax\sum_{n=1}^{N}sim\left(f\left(x_{n}\right),w_{bn}^{t}\right)$$

(17) $$\omega_{bn}^{t}=\sum_{i=1}^{n_{bn}^{t}}f\left(x_{bn}\right)/n_{bn}^{t}$$

By adapting the classifier to the novel classes while keeping the backbone unchanged, it can maximize the preservation of previously acquired knowledge.

### 3.3 Loss function

In this paper, the loss functions consist of three parts: cross-entropy loss (Equation 18), contrastive loss (Equation 19), and feature augmentation loss (Equation 20). The model leverages sufficient data within basic classes to obtain multi-semantic aggregated information from fine-grained images by optimizing per-sample loss, simultaneously, maximizing inter-class margins.

For the extension of frequency-aware guided alternations, the anchor image $x_{i}$ is processed by the cross-entropy loss that computes class features to their targets. It is computed by Equation 18:

(18) $$L_{ce}\left(x_{i}\right)=-\sum_{i}^{b}y_{i}\log\left(p\left(x_{i}\right)\right)$$

To generate the query view $x_{q}$ and the key view $x_{k}$, we apply specific data augmentations. For a specific anchor index i, we define the set of all indices in the current batch (or memory queue) as A(i). We strictly categorize these indices into two subsets: Positive Set: This set contains the indices of samples that share the same class label as the anchor i: $Q_{i}=\left\{j\in A\left(i\right)|y_{j}=y_{i},j\neq i\right\}$. Negative Set: This set contains the indices of samples from all other classes: $K_{i}=\left\{k\in A\left(i\right)|y_{k}\neq y_{i}\right\}$. And we adopt the InfoNCE loss (Ahmed et al., 2024) as our contrastive objective. The objective is to maximize the similarity between the anchor and its positive peers while minimizing the similarity with negative samples. The loss for anchor $x_{i}$ is formulated as Equation 19:

(19) $$L_{cl}\left(x_{i}\right)=\frac{-1}{\left|Q_{i}\right|}\sum_{x_{j}\epsilon Q_{i}}\log\frac{exp\left(x_{i}⊙x_{j}/\tau \right)}{\sum_{x_{k}ЄK_{i}}exp\left(x_{i}⊙x_{k}/\tau \right)}$$

where ⊙ denotes the dot product, $\left|Q_{i}\right|$ is the cardinality of the positive set, and τ is the temperature parameter (set to 16). The denominator sums over all contrastive samples to strictly regulate the embedding space. $x_{j}$ is the element of positive set $Q_{i}$, $x_{k}$ is the element of negative set $K_{i}$. It aims to pull $x_{j}$ closer to $x_{i}$, and push $x_{k}$ further to $x_{i}$. In this paper, to complement contrastive loss, global feature augmentation loss is computed for sample $x_{i}$ to improve the generalization of class separation. Denote the prototype for each class as $P_{j}$, the $L_{Al}$ is expressed by Equation 20:

(20) $$L_{Al}\left(x_{i}\right)=-\frac{1}{R}\sum_{i}^{R}P_{j}\log\left(p\left(x_{i}\right)\right)$$

where R is the number of training images. And the overall training objective can be concluded as Equation 21:

(21) $$L_{loss}=L_{ce}+L_{cl}+L_{Al}$$

## 4 Experimental results and discusses

### 4.1 Dataset

To validate the generalization of our method, additional experiments are conducted on two publicly available herb datasets, with comparisons made against other methods.

**Figure 5:**
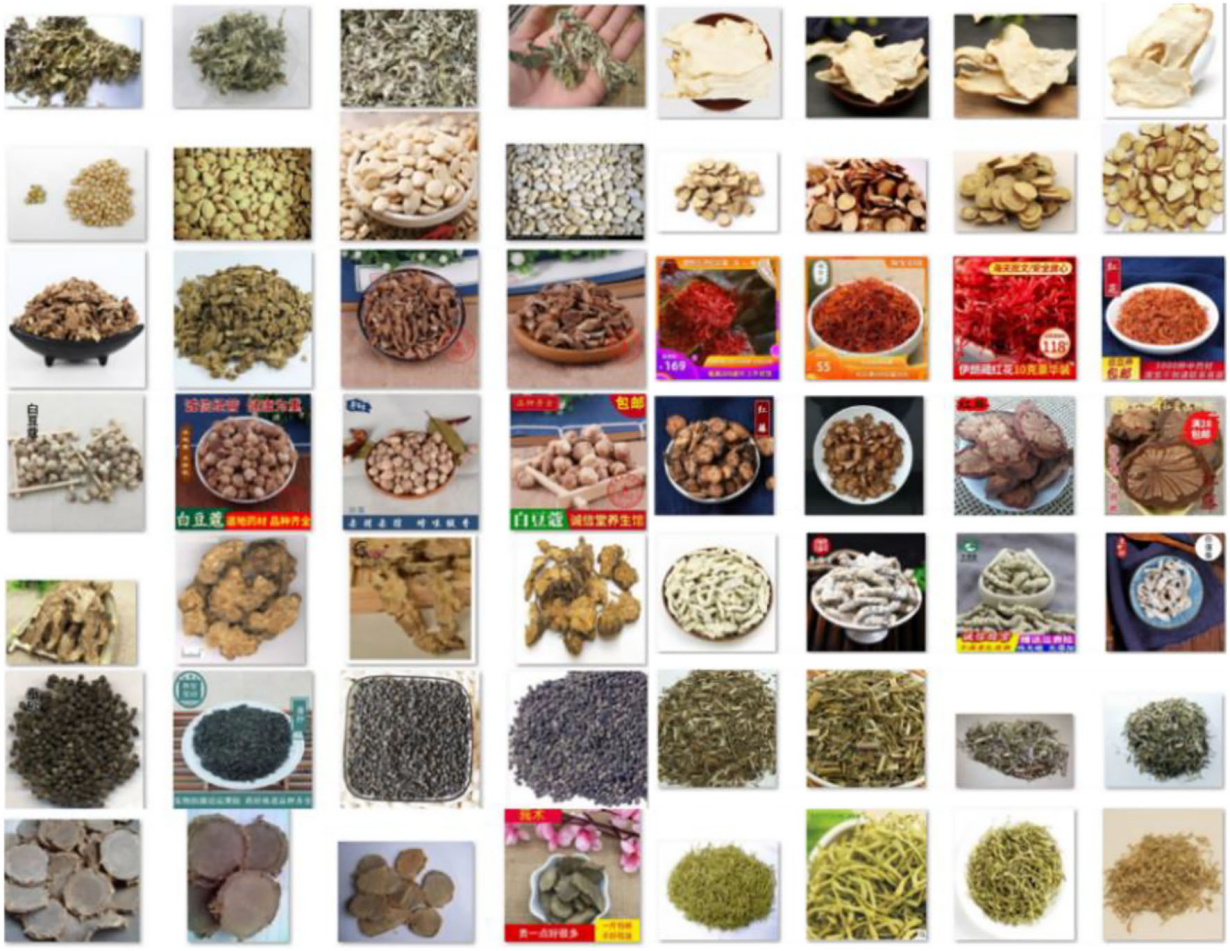
The part of the Chinese Medicine dataset. The Chinese Medicine dataset comprises 20 different types of Chinese medicinal plants, comprising a total of 3000 images.

**Figure 6:**
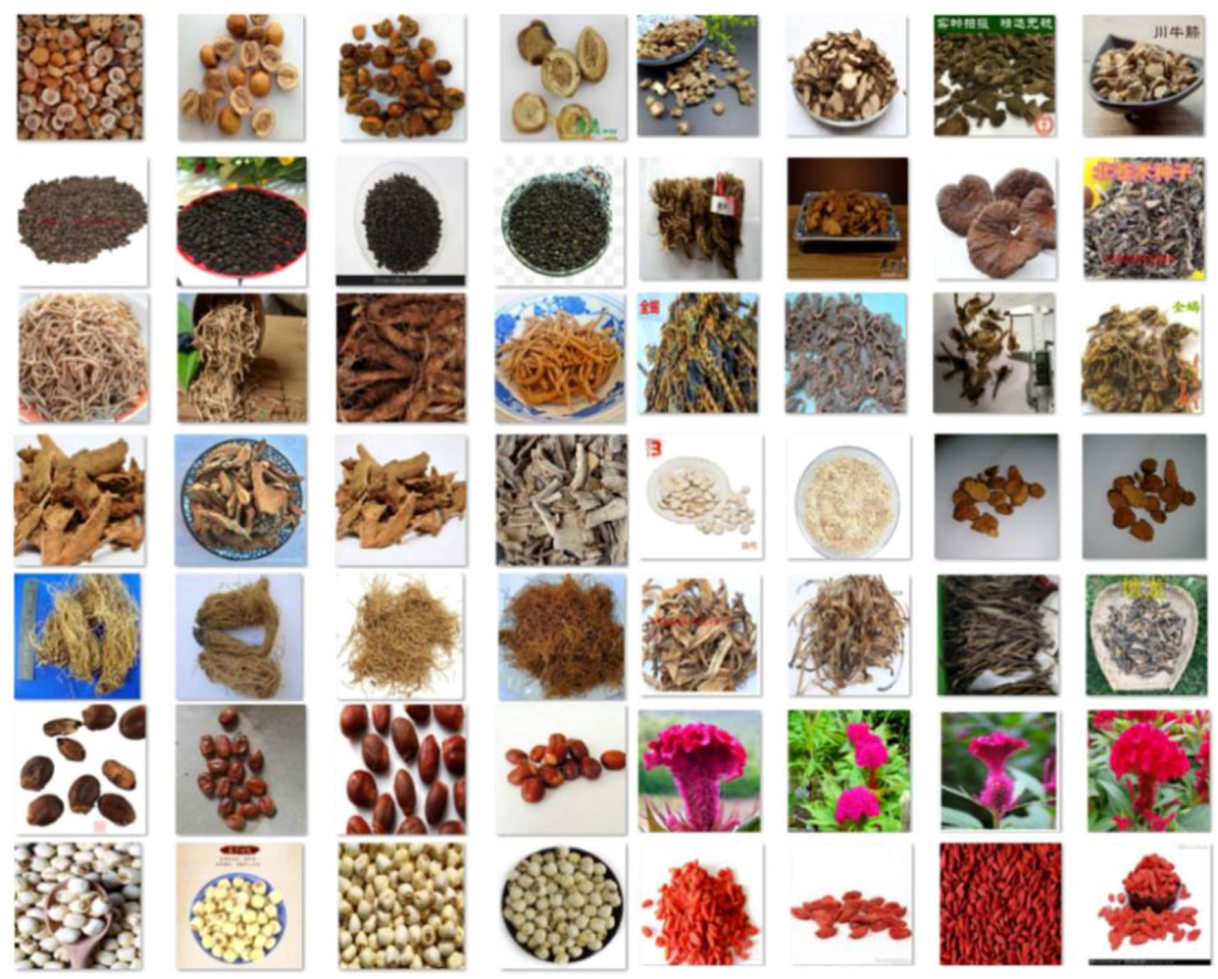
The part of Medicinal Leaf dataset. The Medicinal Leaf dataset contains 100 types of herbal plants, comprising a total of 10000 images.

The two herb datasets are the Chinese Medicine dataset (Thella and Ulagamuthalvi, 2021) and Medicinal Leaf (Huang and Xu, 2023), and excerpts of these datasets are illustrated in **Figures 5** and **6**.

### 4.2 Implementation details

The model is optimized by SGD with 0.9 momentum. The initial learning rate is 0.1, and the learning rate decay strategy is StepLR. The batch size is set to 16, and the final model is obtained when reaches 100 epochs in the basic learning phase. For the incremental learning phase, we fine-tune the per-trained model, and the novel query samples are compared with the key embedding of basic training. The model updates the classifier over 10 epochs to mitigate overfitting. The code is built by using PyTorch=2.2.1 with Python=3.11. The model is trained on a PC (equipped with an Intel i7 processor) with a graphics processing unit card (NVIDIA 4090, 24G memory).

### 4.3 Performance metrics

We use Accuracy, *Precision*, *Recall*, *Specificity*, and *F* 1*Sore* as evaluation metrics as shown in Equations 22–27. Furthermore, harmonic mean value (HM) (Kim et al., 2023; Yang et al., 2023) is used to balance the inherent biases of basic classes with incremental classes.

(22) $$Acc=\frac{TP+TN}{TP+FN+FP+TN}$$

(23) $$\text{Precision}=\frac{TP}{TP+FP}$$

(24) $$\text{Recall}=\frac{TP}{TP+FN}$$

(25) $$\text{Specificity}=\frac{TN}{FP+TN}$$

(26) $$\text{F}1\text{ Score}=\frac{2\times \text{Precision}\times \text{Recall}}{\text{Precision}+\text{Recall}}$$

(27) $$HM=\frac{2\times A_{base}\times A_{inc}}{A_{base}+A_{inc}}$$

where TN represents the number of True Negative, and TP denotes the number of True Positive. FN indicates the number of False Negative, and FP is the number of False Positive. $A_{base}$ is the accuracy of base classes, $A_{inc}$ is the top-1 accuracy of incremental classes.

### 4.4 Performance of identification results

#### 4.4.1 Performance of model identification

We split our dataset into the training set and the testing set. Specifically, 75% of the data is used for training, and the remaining 25% is used for testing. The basic training phase is composed of 16 classes with 200 samples. And there are 4 incremental sequences, within 3 classes and 5 samples of each class. The experimental results of each class are detailed in **Table 1**.

**Table 1:** The experimental results of different varieties by our model.

Class	Precision	Recall	Specificity	F1_ score	Class	Precision	Recall	Specificity	F1_ score
A	100.0	100.0	100.0	100.0	O	97.000	636.0	99.900	76.800
B	82.400	82.400	99.200	82.400	P	100.0	20.000	100.0	33.300
C	100.0	99.600	100.0	99.800	Q	57.900	62.000	98.300	59.900
D	90.200	96.000	99.500	93.000	R	45.800	84.500	96.200	59.400
E	100.0	100.0	100.0	100.0	S	88.900	76.000	99.600	81.900
F	95.800	99.200	99.800	97.500	T	30.300	62.000	96.000	40.700
G	80.900	82.800	99.100	81.800	U	87.500	70.000	99.700	77.800
H	98.600	87.600	99.900	92.800	V	45.000	30.000	99.000	36.000
I	95.800	92.000	99.800	93.900	W	30.000	100.0	95.700	46.200
J	99.500	80.800	100.0	89.200	X	32.300	62.000	97.600	42.500
K	84.800	94.000	99.200	89.200	Z	66.700	2.000	100.0	3.900
L	86.000	93.200	99.300	89.500	Z1	72.200	52.000	99.800	60.500
M	80.700	87.200	99.000	83.800	Z2	91.700	44.000	100.0	59.500
N	93.900	49.200	99.800	64.600	Z3	91.300	42.000	100.0	57.500

**Figure 7:**
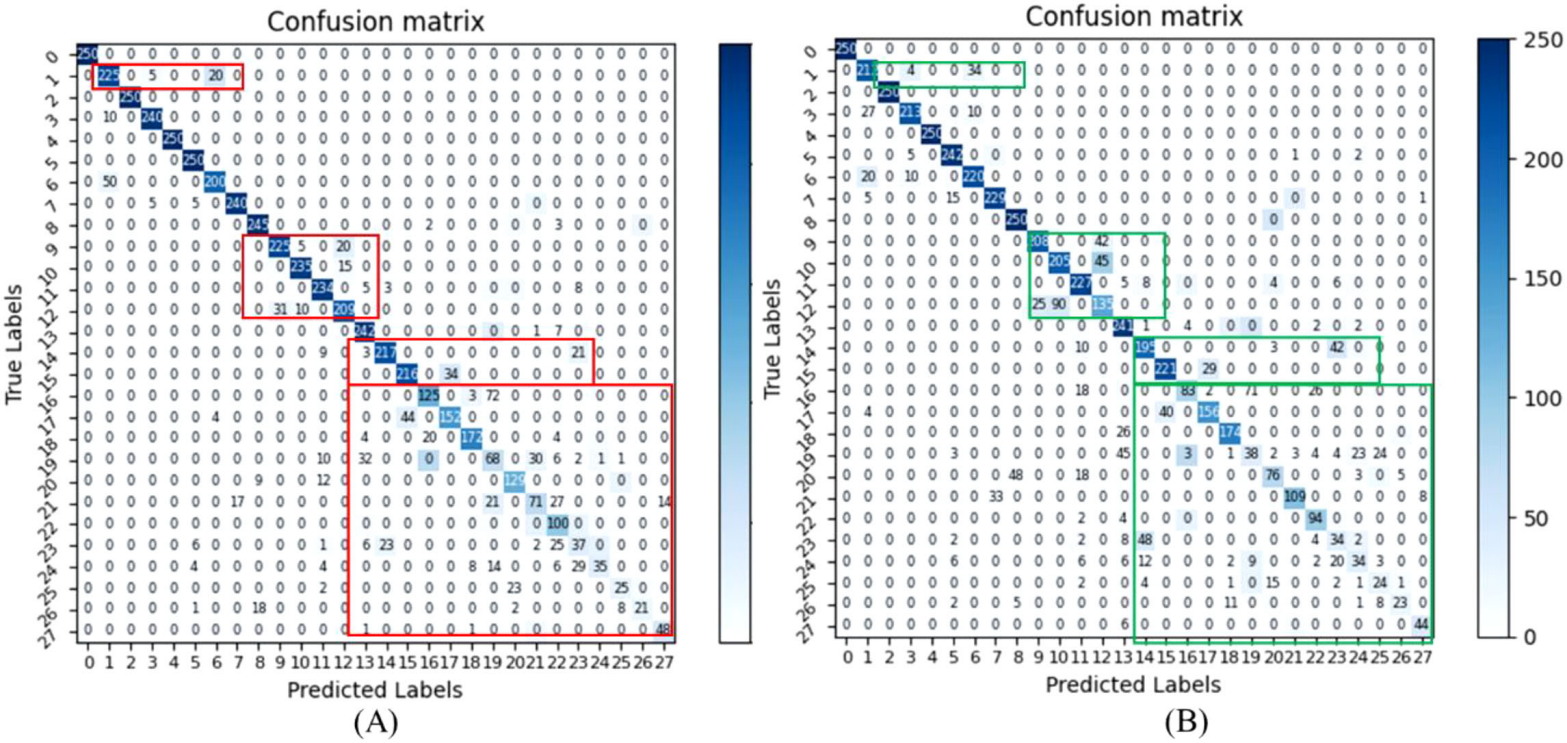
The experimental results of the confusion matrix. **(A)** is ours, **(B)** is the result without high-frequency and low-frequency enhanced images. Significant contrast areas are marked with red and green.

As shown in **Table 1**, the identification performance is notably strong for the base classes. In particular, the model demonstrates high accuracy and robustness in classifying classes A, E, C, F, D, H, and I. In contrast, the F1-scores for the incremental classes predominantly range between 0.3 and 0.6, highlighting the model’s limited effectiveness in learning new classes. For instance, classes R and Q exhibit high recall but low precision; this can be attributed to significant inter-class feature similarity, which leads to misclassification. To further evaluate our model’s performance, we calculated the confusion matrix, and the experimental results are depicted in **Figure 7**.

While the identification results for base classes are comparable across methods, the improvements in incremental classes are more pronounced. In the confusion matrices, a brighter diagonal indicates higher identification accuracy, with significant contrast areas highlighted in red and green. As shown in **Figure 7A**, for base class identification, the marked areas demonstrate that FGDE achieves superior performance with fewer misclassifications compared to other methods. When comparing **Figure 7A** with **Figure 7B**, our model demonstrates distinct advantages over the baseline lacking high-frequency and low-frequency enhancement, particularly in the highlighted regions. Mechanistically, low-frequency components capture global structural features, while high-frequency components extract local fine-grained details, thereby enriching the image representation. Conversely, while the method without frequency-aware extension maintains a visible diagonal for base classes, it performs poorly on novel classes. In contrast, our method exhibits robust performance, indicating that it effectively adapts to novel classes without disrupting previous decision boundaries.

#### 4.4.2 Different losses of model identification

**Figure 8:**
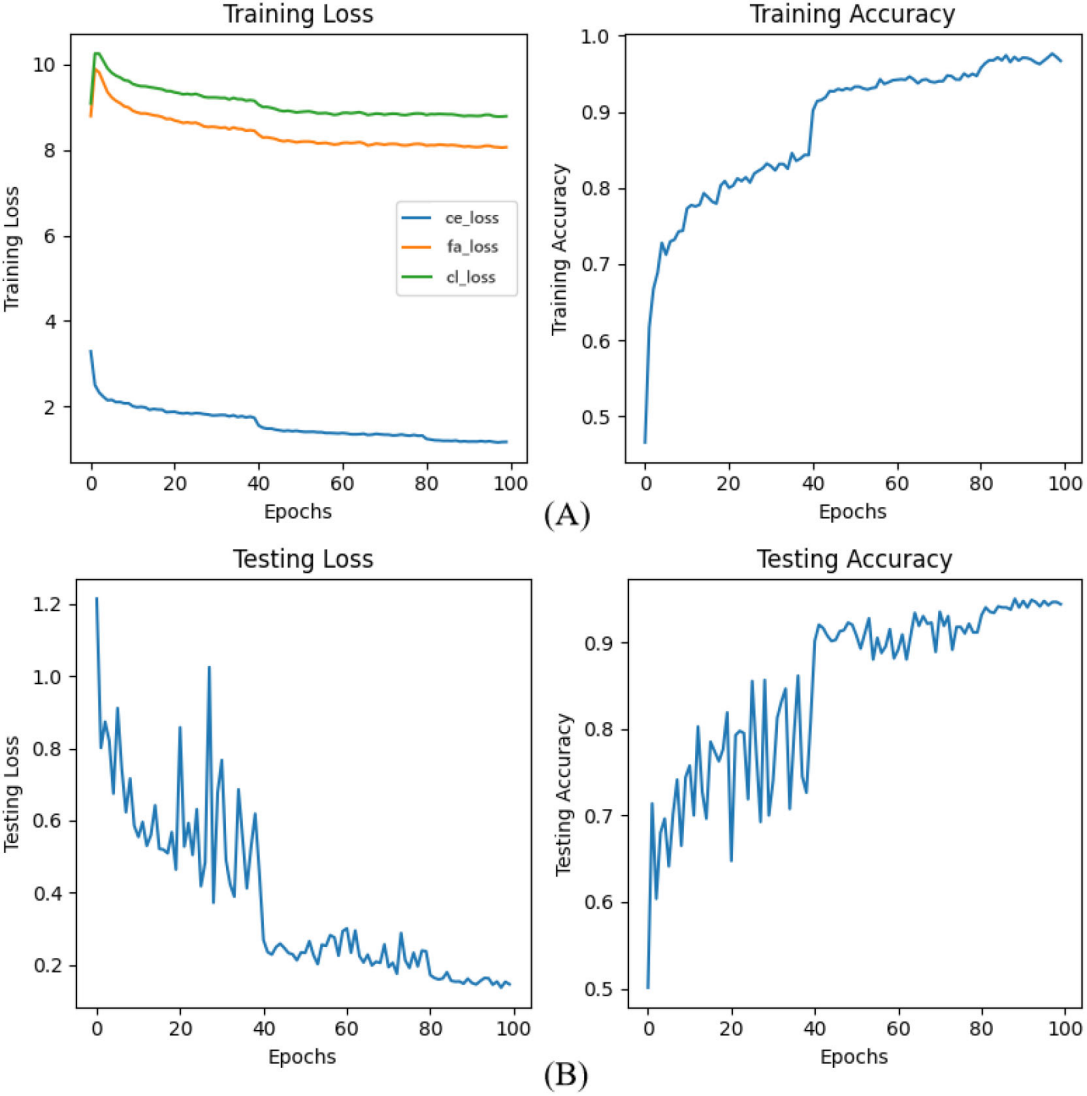
The loss and accuracy results of our model. **(A)** is the curve of different loss changes, and the curve for training accuracy. **(B)** is the curve of the testing loss and the curve of the testing accuracy of our model.

To better evaluate the performance of the model and enhance the explanation of training, the loss and accuracy results of our model are shown in **Figure 8**.

The loss curves and their convergence trends are illustrated in **Figure 8**. Throughout the training process, the loss consistently decreased while accuracy improved, eventually leading to model convergence. Specifically, the trajectories of the multi-objective losses are detailed in **Figure 8A**. The loss exhibits a steady decline until stabilizing at approximately 80 epochs, with the model achieving peak accuracy at epoch 88. Evaluations on the testing set confirm the model’s robust classification performance.

**Figure 9:**
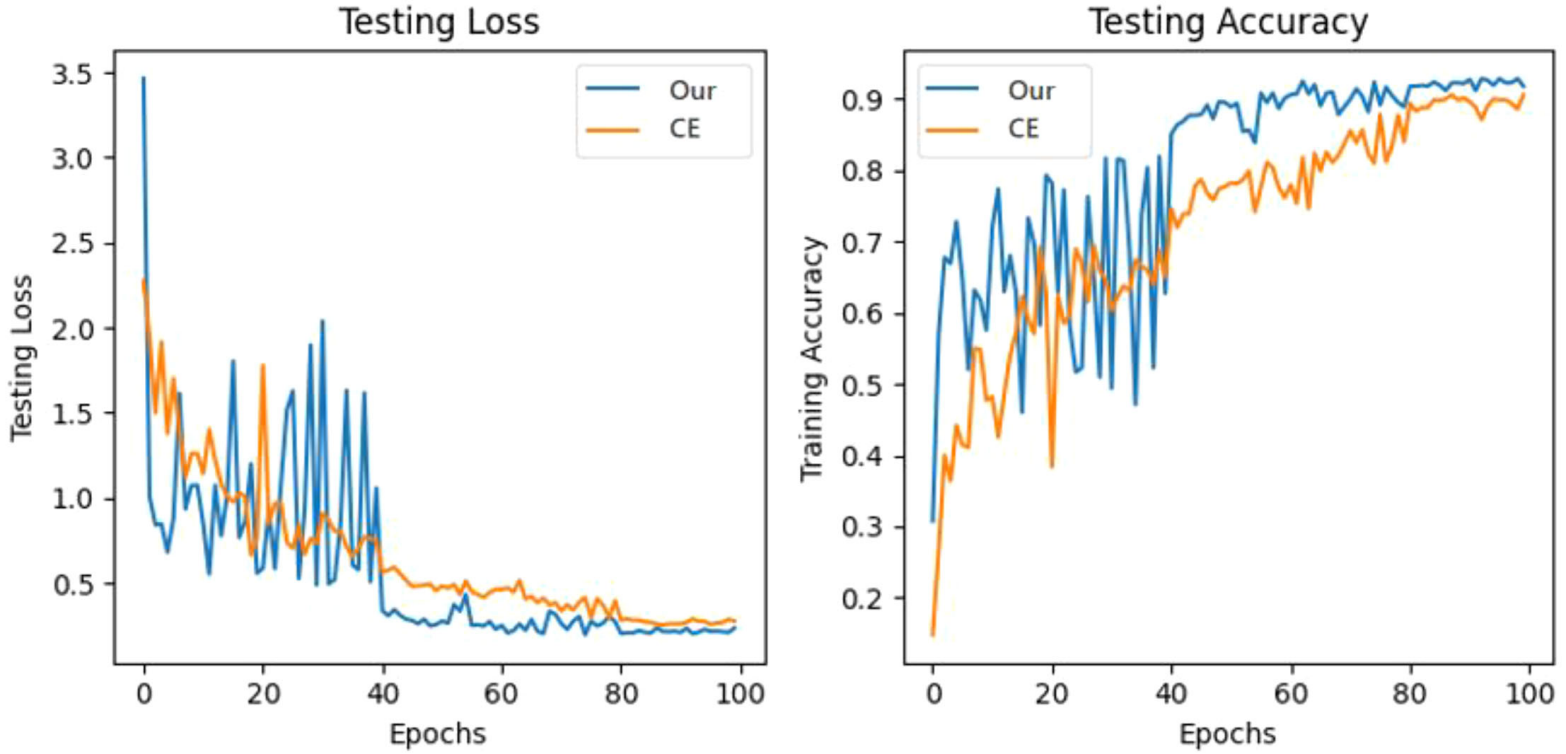
The testing loss and accuracy results of our model and with only CE loss.

Simultaneously, as illustrated in **Figure 9**, While the CE loss baseline provides marginal class separation, our proposed method demonstrates superior capability in distinguishing base classes and integrating novel classes with minimal feature overlap.

#### 4.4.3 Visualization of class separation

**Figure 10:**
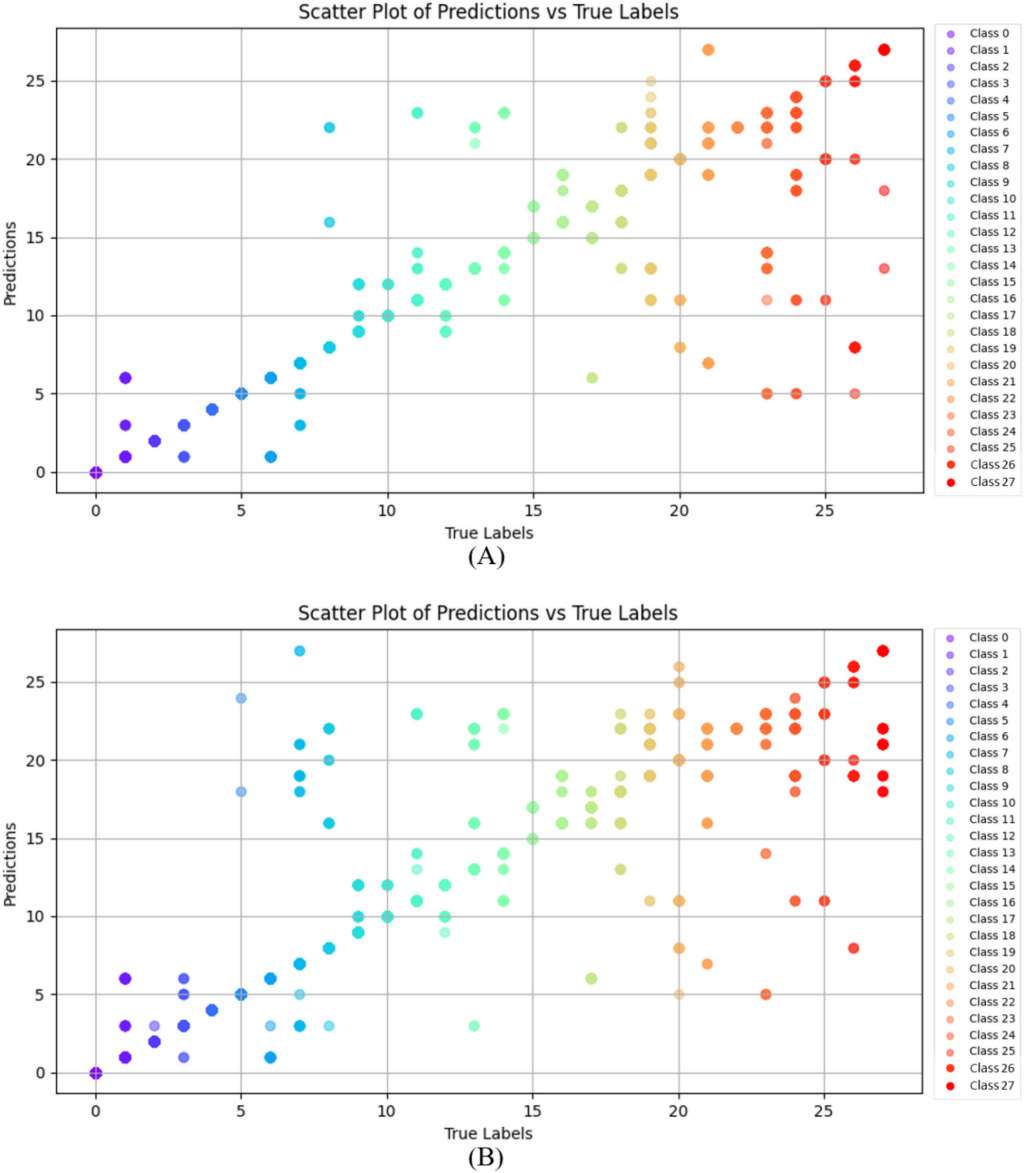
The scatter plot of different classes. **(A)** is our model, and **(B)** is without high-frequency and low-frequency enhanced images. The horizontal axis represents the true labels (True Labels), and the vertical axis represents the predicted labels (Predictions). The color of each point represents a different class, and each color uniquely corresponds to a class.

To verify the effectiveness of our model, we visualize the identification results using a scatter plot, as shown in **Figure 10**. The horizontal axis represents the True Labels, while the vertical axis denotes the Predicted Labels. In this visualization, points aligned closely with the diagonal indicate accurate classification performance. Conversely, points deviating from the diagonal represent misclassifications, where the magnitude of deviation highlights the discrepancy between the predicted and ground truth labels.

In **Figure 10A**, the points are predominantly clustered along the diagonal, indicating that the model achieves robust overall classification performance. In contrast, while **Figure 10B** exhibits some alignment with the diagonal, a significantly larger number of points deviate from it. This dispersion is particularly pronounced in specific categories, such as classes 7 and 8, indicating higher misclassification rates. For the newly added classes, **Figure 10A** maintains relatively high accuracy despite occasional errors. Conversely, **Figure 10B** reveals a marked decline in performance for these later classes, evidenced by a substantial increase in misclassified points and greater deviations from the diagonal. In summary, our method leverages a frequency-based separation strategy: low-frequency components extract fundamental structural features, while high-frequency components capture fine-grained details. This approach enhances inter-class separability and minimizes the interference of new classes on existing representations. Consequently, our method demonstrates significant advantages, exhibiting superior accuracy and robustness.

### 4.5 Comparison with state of the arts

#### 4.5.1 Our dataset

To evaluate the accuracy performance of our model, the state-of-the-art FSCIL models are compared with ours. The experimental results are shown in **Table 2**.

**Table 2:** The results of the comparison of the state-of-the-art FSCIL models.

Methods	Base Accuracy	Accuracy in each sequence (%)				HM (%)	Improve (%)
		1	2	3	4		
iCaRL (Rebuffi et al., 2017)	70.542	67.490	60.371	56.423	50.812	58.774	+27.825
FSLL (Mazumder et al., 2021)	91.421	87.310	84.037	77.034	69.532	75.023	+11.576
C-FSCIL (Hersche et al., 2022)	92.040	85.403	82.760	75.351	70.130	78.411	+8.188
FACT (Zhou et al., 2022)	94.051	87.494	83.057	76.157	70.426	79.284	+7.315
SAVC (Song et al., 2023)	94.081	90.014	82.729	79.747	75.071	84.328	+2.271
Wang (Wang et al., 2024)	94.132	88.093	83.282	76.421	73.579	83.101	+3.498
**Our**	**95.000**	**91.701**	**84.873**	**82.516**	**78.906**	86.599	

The bold values indicates the optimal solution among all methods.

Our proposed method achieves a peak accuracy of 95.000% on the base classes, surpassing all existing baselines. Across subsequent incremental sessions, our method maintains robust performance, recording accuracies of 91.701% in the first session and 78.906% in the fourth. Notably, catastrophic forgetting is significantly better mitigated compared to competing approaches.

While most methods suffer performance degradation as new classes are introduced, distinct patterns emerge. iCaRL exhibits the most inferior performance, starting with a base accuracy of 70.542% and declining rapidly, resulting in the lowest Harmonic Mean (HM) of 58.774%. Although FSLL, FACT, and C-FSCIL achieve respectable base accuracies (91.421%, 92.040%, and 94.051%, respectively), they experience sharp drops in later sessions, yielding HM scores below 80%. SAVC and Wang’s method demonstrate relatively stronger resilience, with HMs of 84.328% and 83.101%, respectively. Nevertheless, our approach consistently outperforms these leading methods across all sessions, securing the highest HM of 86.599%.

This superior performance is attributed to our frequency decomposition strategy. By separating images into low-frequency components (capturing global structural features) and high-frequency components (preserving fine-grained details), we generate a detail-enhanced discriminative representation. This mechanism bolsters both base class separability and novel class generalization. In summary, our model retains base class knowledge while adapting to new sequences with minimal degradation, marking a significant advancement over state-of-the-art methods in solving the FSCIL challenge.

#### 4.5.2 Visualization of class activation maps

**Figure 11:**
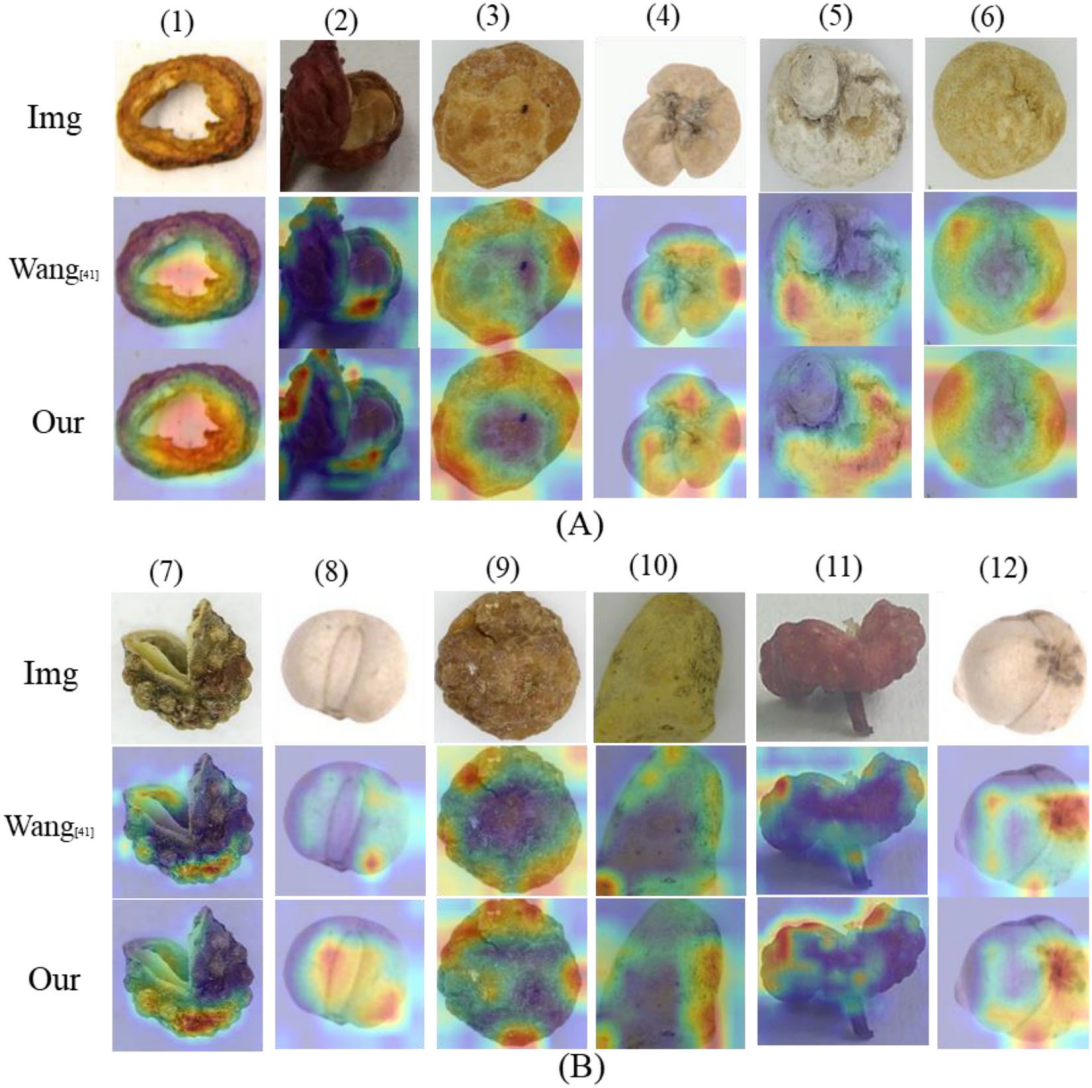
The visualization of the different attention modules for each class. The heat maps of each class are randomly selected. The first is the original image, the second is the heatmap of Wang’s (Wang et al., 2024) method, and the last is ours. The results are organized into **(A, B)**. **(A)** are the base classes, **(B)** are the incremental classes.

Class Activation Maps (CAMs) are essential for interpreting model decisions by highlighting influential image regions. For each instance, the original image is shown with its corresponding CAMs in **Figure 11**. The color intensity represents the activation level, signifying the importance of each region in the model’s classification result.

From **Figure 11**, a systematic comparison across both base and incremental classes reveals a consistent pattern of superior performance by our method. Our approach demonstrates significantly more accurate localization of target objects, with activations that adhere tightly to object boundaries while effectively suppressing background noise. For instance, in items (1), (4), (8), and (10) (Wang et al., 2024), produces diffuse activations that often spill into the background or focus on restricted, peripheral regions. In contrast, our method generates heatmaps that are precisely centered on the targets, covering their salient regions more effectively. Furthermore, our model consistently yields more comprehensive activation maps that encompass the entire object, suggesting the acquisition of a holistic representation. This is particularly evident in items (2), (5), (7), and (11). Whereas (Wang et al., 2024) tends to fixate on local textures or edges, our model captures the full semantic structure of the object. This holistic understanding is crucial for robust classification, rendering the model less susceptible to variations in orientation or partial occlusion. Moreover, the heatmaps generated by our method exhibit a more concentrated focus on the objects’ discriminative regions. In contrast, the activations in the Wang (Wang et al., 2024) model appears scattered and less intense, as evident in examples (5), (9), and (12). Our model, conversely, produces strong, focused activations localized on the core features of the objects. This indicates that our approach more effectively identifies key predictive features and is less prone to relying on spurious image correlations. These qualitative results strongly support our hypothesis that the proposed methodology facilitates learning more robust and interpretable feature representations. By generating more complete and accurately localized heatmaps, our model demonstrates a deeper semantic understanding of the image content and enhanced mitigation of catastrophic forgetting.

#### 4.5.3 Chinese medicine dataset

**Figure 12:**
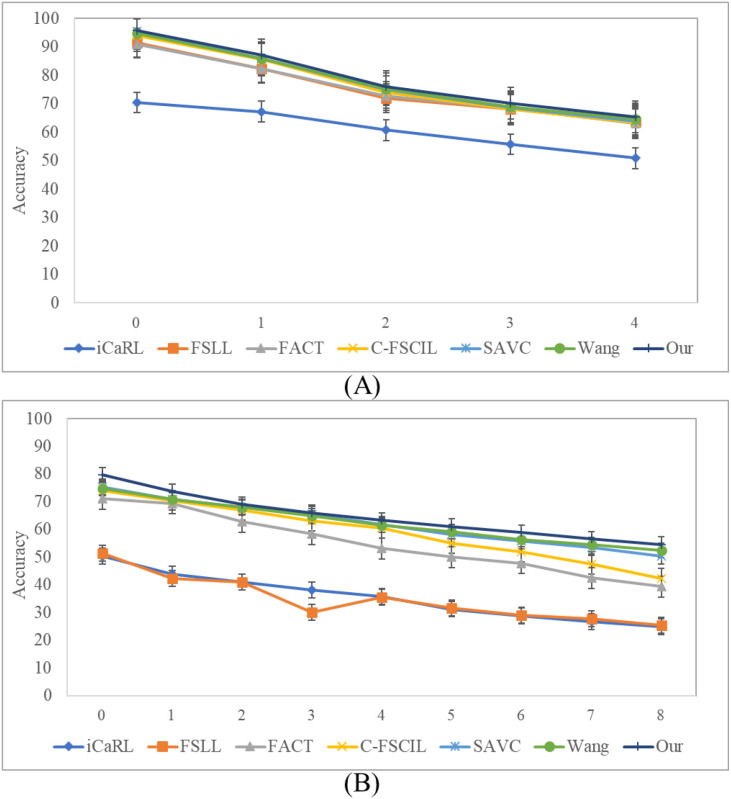
Comparison with the state-of-the-art on two public datasets: **(A)** Chinese Medicine and **(B)** Medicinal Leaf. Error bars indicate the standard deviation, which are used to visualize the performance variance and stability of each method.

We compare our method with other state-of-the-art methods on the Chinese Medicine dataset, and the comparison results are shown in **Figure 12**. From **Figure 12A**, the base classes are 8, the number of classes in each incremental subsequent is 4, and the N-way is set to 3. We can see that our model exhibits the highest accuracy among other mainstream methods. From **Figure 12B**, the model is initialized with 60 base classes, followed by incremental sessions containing 8 classes each, with the setting of 3-shot samples per class.

As observed in **Figure 12**, iCaRL exhibits the most precipitous performance decline, suggesting that its replay-based strategy lacks the stability required for FSCIL tasks and struggles to mitigate interference from novel classes. FSLL attains high initial accuracy but suffers a sharp drop in later stages, indicating that despite early effectiveness, its long-term generalization capability is limited. FACT and C-FSCIL display a more gradual decay, reflecting stronger knowledge retention, although performance degradation persists. Conversely, SAVC and the method by Wang et al. maintain relatively stable performance, particularly in later sessions. Furthermore, to evaluate the statistical reliability of our results, we add the error bars to represent the standard deviation in **Figure 12**. Our method is consistently compact for incremental sessions in both Figures (A) and (B). This low variance indicates that our FGDE model is highly robust to initialization differences and data sampling fluctuations, maintaining stable performance even as the number of classes increases. In contrast, baseline methods such as iCaRL and FSLL exhibit larger error bars in several sessions (e.g., Session 0 and 3 in **Figure 12B**), suggesting higher instability. Notably, our method outperforms all competing approaches, achieving consistent improvement and minimal degradation. By leveraging fine-grained feature comparison and multi-semantic discrimination, our approach significantly enhances adaptability to novel categories while effectively mitigating catastrophic forgetting.

### 4.6 Horizontal comparison and performance trade-offs

#### 4.6.1 Results of different backbones

To select the appropriate backbone to extract features in our model, the various backbones are compared to reflect the impact of model efficiency. We also evaluate the model with a hybrid transformer structure, such as MBConv (Howard et al., 2017).

Using running time as a key feasibility metric, we compared various backbones in **Table 3**. ResNet20 and ResNet50 showed lower accuracy and longer inference times compared to ResNet18. VGG’s simpler architecture limited its feature extraction, reducing base class accuracy, while EfficientNet and DenseNet121 proved computationally expensive due to their complexity. Results from MBConv further highlighted the limitations of hybrid transformer structures in this setting. Although VGG19 improved incremental class accuracy by 1.322%, its slower running time made it less viable. Thus, ResNet18 was selected for achieving the highest comprehensive performance.

**Table 3:** The results of the comparison of the different backbones.

Backbone	Base accuracy	Accuracy in each sequence (%)				HM (%)	Improve (%)	Time (min)
		1	2	3	4			
VGG-16 (Simonyan and Zisserman, 2014)	70.250	67.365	64.439	62.742	60.474	63.755	+22.844	251
VGG-19 (Simonyan and Zisserman, 2014)	90.865	89.423	**88.702**	**86.899**	**86.659**	**87.921**	-1.322	292
ResNet20 (He et al., 2016)	86.875	71.875	72.132	68.275	65.767	69.512	+17.087	153
ResNet50 (He et al., 2016)	93.510	86.096	82.841	74.361	72.879	79.044	+7.555	198
EfficientNet-B0 (Tan, 2019)	90.625	84.352	81.944	76.236	73.377	78.977	+7.622	231
DenseNet121 (Huang et al., 2017)	90.745	79.973	76.389	71.213	70.911	74.622	+11.977	375
MBConv (Howard et al., 2017)	88.094	84.057	78.157	75.426	70.361	77.000	+9.599	652
**ResNet18** (He et al., 2016)	**95.000**	**91.701**	84.873	82.516	78.906	86.599		**131**

The bold values indicates the optimal solution among all methods.

#### 4.6.2 Results of different number of classes

To evaluate the learning ability and generalization ability of our model to incremental classes, experiments with different numbers of classes are performed for the basic training. This paper compares the base 12 classes with the 16 classes. When the base classes are 12, the number of classes in each incremental subsequent is 4, and N-way is set to 4. When the base classes are 16, the number of classes in each incremental subsequent is 3, and N-way is set to 3. The experimental results are shown in **Table 4**.

**Table 4:** The results of the comparison of the different numbers of classes.

Base classes	Base accuracy	Accuracy in each sequence (%)				HM (%)	Improve (%)
		1	2	3	4		
12	**96.875**	81.971	74.375	74.041	65.98	74.092	+12.507
16	95.000	**92.509**	**86.071**	**80.234**	**79.072**	**86.599**	

The bold values indicates the optimal solution among all methods.

For base 12 classes, the base accuracy is a better 1.875% than the 16 classes. However, base 12 classes perform 10.380% worse than base 16 classes in the incremental sequences. The experimental results demonstrate that a greater number of base classes enhances the ability of the model to learn and capture fine-grained features more effectively. Finally, we set the base class to 16, N-way is 3.

#### 4.6.3 Results of different sizes of cropping

In the original settings, images are initially cropped to enrich the fine-grained feature space and increase image diversity. To evaluate the impact of cropping sizes on model performance, our experiment examines the different cropping sizes. The comparison results are shown in **Table 5**.

**Table 5:** The results of the comparison of the different sizes of cropping.

Crop size	Base accuracy	Accuracy in each sequence (%)				HM (%)	Improve (%)
		1	2	3	4		
**128**	**95.000**	**92.509**	**86.071**	**80.234**	**79.072**	**86.599**	
96	92.909	85.849	81.771	75.184	71.956	78.690	+7.909
64	90.625	84.352	81.944	76.236	73.377	78.977	+7.622
32	90.745	79.973	76.389	71.213	70.911	74.622	+11.977

The bold values indicates the optimal solution among all methods.

From **Table 5**, it is evident that crop size significantly affects feature diversity. The 128-crop size achieves the highest discrimination, surpassing the 96-crop and 32-crop settings by 7.909% and 11.980%. Additionally, the similar HM scores for crop sizes 96 and 64 suggest a consistent feature distribution at these scales. Given these results, we fixed the crop size at 128 to ensure optimal model performance.

#### 4.6.4 Results of different numbers of base sequences

To verify the effectiveness of our networks for few-shot images, we also compared the impact of different numbers of base classes on model performance. To ensure fairness, the remaining parameters remain unchanged in the comparison. The experimental results are shown in **Table 6**.

**Table 6:** The results of the comparison for the different numbers of base sequences.

Number	Base accuracy	Accuracy in each sequence (%)				HM (%)	Improve (%)
		1	2	3	4		
300	**95.312**	88.916	85.822	76.565	72.423	80.932	+5.667
250	**95.533**	86.809	82.350	72.891	70.201	78.063	+8.536
200	95.000	**92.509**	**86.071**	**80.234**	**79.072**	**86.599**	

The bold values indicates the optimal solution among all methods.

The results indicate that the accuracy for base classes remains relatively stable regardless of the class count. However, regarding the identification of incremental classes, the configuration with 200 base classes yields an accuracy 8.536% higher than that with 250 base classes and 5.667% higher than that with 300 base classes. These findings suggest that 200 base classes offer the optimal balance between feature diversity and model generalization.

## 5 Conclusion

In this paper, we proposed the FGDE address the challenge of FSCIL within the context of fine-grained images. This method innovatively synergizes the low-frequency and high-frequency components with dual-domain contrastive learning to enhance feature discriminability. Unlike existing methods that struggle with subtle inter-class differences, our approach effectively sharpens decision boundaries while maintaining the stability of base classes. Extensive experiments on both proprietary and public TCM datasets demonstrate that FGDE outperforms state-of-the-art methods, offering a robust solution for balancing plasticity and stability. In future work, we aim to further refine identification performance for highly fine-grained categories. Promising directions include integrating diffusion models to address data imbalance via high-fidelity sample generation and employing Graph Convolutional Networks (GCNs) to capture neighborhood structures, thereby further alleviating catastrophic forgetting.

## Data Availability

The original contributions presented in the study are included in the article/supplementary material. Further inquiries can be directed to the corresponding author.
